# Ultra-processed foods: a fit-for-purpose concept for nutrition policy activities to tackle unhealthy and unsustainable diets – ADDENDUM

**DOI:** 10.1017/S0007114523000016

**Published:** 2023-05-28

**Authors:** Mark Lawrence

Declaration of interest information was not included in the above published article. A Declaration of Interest section has now been added (as shown below), and the online PDF and HTML versions updated.

“**Declaration of Interest**

The author is a Board member of Food Standards Australia New Zealand. The views expressed in this article do not necessarily reflect the positions of any organisation with which he is associated. The author receives funding from the Australian Research Council Discovery Project; DP190101323, ‘Reforming evidence synthesis and translation for food and nutrition policy’. He declares that there are no conflicts of interest.”
